# Selective nonoperative versus operative management of liver gunshot injuries: a retrospective cohort study

**DOI:** 10.1308/rcsann.2022.0061

**Published:** 2024-05-24

**Authors:** RR Dalcin, YTM Petrillo, LAC Alves, MK Fonseca, AS Almeida, CO Corso

**Affiliations:** ^1^ Federal University of Rio Grande do Sul (UFRGS), Porto Alegre, Brazil; ^2^ Hospital de Pronto Socorro de Porto Alegre, Brazil; ^3^ Hospital Nossa Senhora da Conceição, Porto Alegre, Brazil

**Keywords:** Abdominal injuries, Liver, Penetrating wounds, Liver/surgery

## Abstract

**Introduction:**

Experience accumulated over the last decades suggests nonoperative management (NOM) of civilian gunshot liver injuries can be safely applied in selected cases. This study aims to compare the outcomes of selective NOM versus operative management (OM) of patients sustaining gunshot wounds (GSW) to the liver.

**Methods:**

A registry-based retrospective cohort analysis was performed for the period of 2008 to 2016 in a Brazilian trauma referral. Patients aged 16–80 years sustaining civilian GSW to right-sided abdominal quadrants and liver injury were included. Baseline data, vital signs, grade of liver injury, associated injuries, injury severity scores, blood transfusion requirements, liver- and non-liver-related complications, length-of-stay (LOS), and mortality were retrieved from individual registries.

**Results:**

A total of 54 patients were eligible for analysis, of which 37 underwent NOM and 17 underwent OM. The median age was 25 years and all were male. No statistically significant differences were observed between groups regarding patients’ demographics, injury scores, grade of liver injury and associated lesions. NOM patients tended to sustain higher-grade injuries (86.5% vs 64.7%; *p* = 0.08), and failure of conservative management was recorded in two (5.4%) cases. The rate of complications was 48% with no between-group statistically significant difference. Blood transfusion requirements were significantly higher in the OM group (58.8% vs 21.6%; *p* = 0.012). The median LOS was seven days. No deaths were recorded.

**Conclusion:**

Patients with liver GSW who are haemodynamically stable and without peritonitis are candidates for NOM. In this study, NOM was safe and effective even in high-grade injuries.

## Introduction

The liver is the most frequently injured organ in abdominal trauma.^1^ Nonoperative management (NOM) is considered the mainstay of treatment for hepatic blunt and stab wounds in haemodynamically stable patients; however, it has yet to achieve widespread acceptance for gunshot injuries to the abdomen.^2^

Mandatory laparotomy has been the traditional approach of choice for these cases, though it may result in approximately one-quarter of negative or nontherapeutic surgical explorations.^3^ Besides potential long-term complications such as bowel obstruction and incisional hernia,^4^ operative management (OM) in penetrating liver injuries also has an increased liver-related complication rate of up to 50%.^1^

Experience accumulated over the last two decades has suggested that NOM of civilian gunshot liver injuries could be safely applied to selected haemodynamically stable patients without peritonitis.^5^ Recent improvements in diagnostic and therapeutic adjunct tools that facilitated widespread implementation of NOM for blunt solid organ trauma, such as computed tomographic (CT) scan, endoscopic, endovascular and percutaneous interventions, have allowed for the evaluation of this approach for penetrating liver trauma.^4^ Previous reports have shown that NOM for gunshot wounds (GSW) to the abdomen is independently associated with decreased mortality and complications compared with OM in appropriately selected patients, even when there is a known solid organ injury.^6^

The present study aims to compare the outcomes of selective NOM versus OM of haemodynamically stable patients sustaining GSW to the liver admitted to a trauma referral hospital in southern Brazil.

## Methods

This is a retrospective cohort analysis of the General and Trauma Surgery database of the Hospital Municipal de Pronto Socorro in Porto Alegre, a trauma centre located in southern Brazil, for the period of 2008–2016. The study population included patients aged 16–80 years sustaining civilian GSW to right-sided abdominal quadrants with liver injury. Haemodynamic instability (defined as systolic blood pressure [SBP] <90mmHg not responding to initial resuscitation), Glasgow Coma Score (GCS) <14 and associated hollow viscus injury were considered exclusion criteria. The severity of liver injury was classified according to the American Association for the Surgery of Trauma and Organ Injury Severity (AAST-OIS), extending from 1 to 6.

Data on demographics, vital signs and GCS at admission, grade of liver injury severity, associated abdominal and extra-abdominal injuries, injury severity scores, blood transfusion requirements, liver- and non-liver-related complications, length-of-stay (LOS) and in-hospital mortality were retrieved from individual registries. Patients were divided into two groups based on the selected management (NOM or OM) for comparison, and further classified according to the AAST grade of injury in minor (AAST I – II), moderate (III) and major (AAST IV–VI). The primary endpoints assessed were LOS and death, and secondary outcomes included failure of NOM, liver-related complications and blood transfusion requirements. Patients were followed-up for 30 days after hospital discharge.

Data were analysed in accordance with STROBE guidelines.^7^ Descriptive statistical analysis was performed for all variables. Pearson chi-square or Fisher’s exact test were applied to compare categorical values where appropriate. Continuous variables were compared using Student’s *t*-test or Mann–Whitney test for parametric and nonparametric data, respectively. The level of significance was set at 5%. Statistics were performed using the IBM SPSS® v21.0. Ethical approval for this study was obtained from the ethics committee of Hospital de Clínicas de Porto Alegre, which is accredited by the Office for Human Research Protections as an Institutional Review Board.

## Results

**Figure 1:**
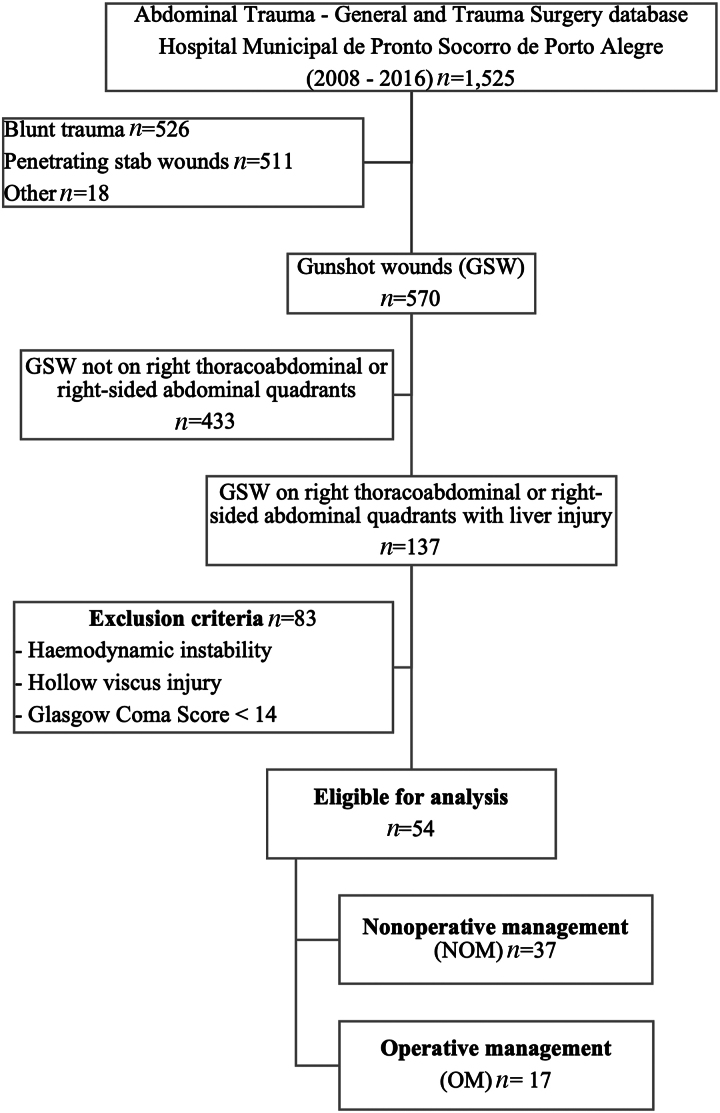
Study design

During the study period, 1,525 trauma victims were admitted to the Division of General and Trauma Surgery of the Hospital Municipal de Pronto Socorro de Porto Alegre. Of these, 137 (9%) sustained penetrating injuries to the right thoracoabdominal or right abdominal quadrants. A total of 54 (3.5%) fulfilled the inclusion and exclusion criteria and were eligible for the analysis, of which 37 underwent NOM and 17 were operated shortly after admission (OM) (Figure 1).

The median age was 25 years (range, 13–60), and all patients were male. The average Revised Trauma Score (RTS) was 7.6 (range, 6.2–7.84). No statistically significant differences were observed between groups regarding patients’ demographics, RTS, grade of liver injury, extra-abdominal GSW and associated lesions (Table 1). The most frequent associated intra-abdominal injury identified in both groups was a laceration to the diaphragm and right kidney. Diaphragm injuries in the NOM group were presumed by the presence of right-sided haemo/pneumothorax or lung contusion from the same bullet trajectory. Associated extra-abdominal injuries were mainly haemo/pneumothoraces, all of which were treated with intercostal chest drains.

**Table 1:** Baseline characteristics of study groups

	NOM (*n* = 37)	OM (*n* = 17)	*p*-value
**Age (years)**	27.5 ± 9.8	27.8 ± 9.9	0.915
**RTS**	7.47 ± 0.34	7.46 ± 0.39	0.958
**Grade of liver injury/AAST (%)**			0.081
Minor	5 (13.5)	6 (35.3)	
I	2 (5.4)	0	
II	3 (8.1)	6 (35.3)	
Moderate			
III	12 (32.4)	5 (29.4)	
Major	20 (54.5)	6 (35.3)	
IV	16 (43.2)	6 (35.3)	
V	4 (10.8)	0	
**Intra-abdominal associated injuries (%)**			
Diaphragm	29 (78.4)	10 (58.8)	0.192
Right kidney	5 (13.5)	4 (23.5)	0.439
Spleen	0	2 (11.8)	0.095
**Extra-abdominal GSW (%)**	8 (21.6)	7 (41.2)	0.192
**Extra-abdominal injuries (%)**	31 (83.8)	12 (70.6)	0.293
Haemo/pneumothorax	27 (73.0)	9 (52.9)	0.254
Bilateral haemo/pneumothorax	3 (8.1)	1 (5.9)	1.000
Lower limb	0	1 (5.8)	1.000

Data presented as mean ± SD or n (%) NOM = nonoperative management; OM = operative management; RTS = Revised Trauma Score; AAST = American Association for the Surgery of Trauma; GSW = gunshot wound.

All NOM patients underwent contrast-enhanced abdominal CT scan to determine the missile trajectory and the grade of liver injury. The AAST-OIS classifications were mainly moderate–major, and NOM patients tended to sustain higher-grade injuries (86.5% *n* = 32 vs 64.7% *n* = 11; *p* = 0.08), with no statistical significance. Grade V lesions were identified only in four cases, all of which were in the NOM group. Liver lacerations among patients undergoing OM were addressed by haemostatic techniques alone, such as electrocoagulation and hepatorrhaphy, with or without placement of abdominal drains according to the attending surgeon’s discretion. Neither case required anatomic or non-anatomic resections, shunting procedures, hepatic vascular isolation or intraoperative packing due to intraoperative haemodynamic instability.

The overall rate of complications was 48% with no between-group statistically significant difference. More than two-thirds of these events were non-liver-related (*n* = 23), including atelectasis, nosocomial pneumonia and empyema. Thoracotomy evacuation of infected chest collections and lung decortication was required in five patients in the NOM group. Complications related to liver injury were observed in three cases (5.5%) and consisted of intra-abdominal abscesses – two in the NOM and one in the OM group. The former was successfully treated with intravenous antibiotics, though one also underwent ultrasound-guided percutaneous drainage. The case in OM group was classified as a Grade III liver injury and required a relaparotomy to completely resolve the abscess. None presented biliary complications such as fistula or biliomas.

Blood transfusion requirements were significantly higher in the OM compared with the NOM group (58.8%; *n* = 10 vs 21.6%; *n* = 8; *p* = 0.012). There was no statistically significant difference in the amount of blood product types (packed red blood cells and fresh frozen plasma) received between groups. The overall median LOS was 7 (2–36) days. There were no deaths and no onset of new complications requiring readmission. Patient outcomes are detailed in Table 2.

**Table 2:** Patient outcomes in study groups

	NOM (*n* = 37)	OM (*n* = 17)	*p*-value
**Complications (%)**			
Pneumonia/atelectasis/empyema	12 (32.4)	7 (41.2)	0.750
Intra-abdominal abscess	2 (5.4)	1 (5.9)	0.230
**NOM failure (%)**	2 (5.4)	–	–
**Blood transfusion (%)**	8 (21.6)	10 (58.8)	0.012
Packed RBCs (units)	2.5 (2–4)	2 (2–2)	0.237
FFP (units)	2 (2–4)	2.5 (2–4)	0.945
Length-of-stay (days)	7 (5–15)	7 (7–12.5)	0.509

Data presented as median (interquartile range) or n (%) NOM = nonoperative management; OM = operative management; RBC = red blood cells; FFP = fresh frozen plasma.

Two cases ultimately failed NOM. Surgical exploration was performed in a patient sustaining a Grade IV liver injury six hours after admission due to an episode of hypotension, and hepatorrhaphy with right diaphragmatic repair were the only therapeutic interventions. The other case refers to a patient with a Grade V liver injury and arterial blush on CT scan that developed progressive abdominal tenderness and distension. A repeated CT scan revealed a large perihepatic haematoma and a moderate amount of free fluid, which prompted an exploratory laparotomy, 24 hours after admission, to achieve haemostasis, evacuate cloths and drain the liver properly. Both subjects had an otherwise unremarkable recovery and were discharged home within two weeks after admission Patient outcomes according to each grade of liver injury are detailed in Table 3.

## Discussion

In the last three decades, the operative management of penetrating liver injuries has been challenged in favour of selective NOM with a significant improvement of outcomes.^1^ An early prospective series conducted by Demetriades *et al*^8^ (1986) reported successful NOM in a selected group of patients sustaining both isolated gunshot and stab wounds to the liver, with no complications or mortality. Subsequently, several authors have suggested that selective NOM for GSW to the abdomen with known liver injury is safe, even for higher-grade lesions requiring adjunctive management, such as angioembolisation.^9–14^

Despite these encouraging results, a major concern in NOM of hepatic GSW remains the potential delay of a needed operation, given the high incidence of associated intra-abdominal injuries or massive bleeding from the injured liver resulting from a high-energy transfer.^6,8,15^ In the present study, with appropriate case selection (haemodynamically stable patients without peritonitis on admission), no increase in mortality or complication rates and no missed hollow viscus injury were reported. Also, the two cases of failed NOM had otherwise favourable outcomes and no further complications related to the delayed laparotomy, which is consistent with previous literature.^1^

It has been previously emphasised that NOM performed in patients sustaining stab wounds to the liver is somewhat comparable to isolated injuries resulting from standard low-velocity weapons and projectiles, as these lesions often do not require repair, and in 40% of cases there is no active bleeding.^9,15,16^ This approach thus contributes to reducing nontherapeutic laparotomy rates and its associated potential complications, such as wound infection, ileus, thromboembolism, incisional hernias and adhesive small bowel obstruction, which can occur in up to 42% of cases.^17,18^ Moreover, the routine exploration of an injured liver may disrupt a contained liver haematoma, resulting in further bleeding. This was confirmed in our study, as blood transfusion requirements were significantly higher in the OM group (58% vs 21%). Demetriades *et al*^8^ reported similar results, with 31% of patients in the OM group requiring blood transfusion against 10% of those undergoing NOM. Starling *et al*^14^ found an overall 10.8% of cases submitted to NOM for GSW with isolated liver injury requiring blood transfusion.

**Table 3:** Patient outcomes according to grade of liver injury

	I (*n* = 2)	II (*n* = 9)	III (*n* = 17)	IV (*n* = 22)	V (*n* = 4)	*p*-value
Complications (%)	1 (50)	2 (22)	5 (29)	12 (54)	2 (50)	0.256
** **Abdominal abscess	0	0	1 (6)	1 (4.5)	1 (25)	0.466
NOM failure (%)	0	0	0	1 (4.5)	1 (25)	0.184
Blood transfusion (%)	0	1 (11)	6 (35.3)	10 (45.5)	1 (25)	0.330
Length-of-stay (days)	3	7 (3–8)	7 (5–13)	11 (7–19)	9 (7–12)	0.388

Data presented as median (interquartile range) or n (%) NOM = nonoperative management

NOM is also reported to be safe and effective in high-grade liver injuries given the appropriate clinical setting.^9,17,19^ Coccolini *et al*^1^ advocate that the proper indication of NOM must consider the AAST classification, the patient’s haemodynamic status and associated injuries, according to the classification proposed by the World Society of Emergency Surgery (WSES). In our study, NOM was successfully conducted in 95% of cases, of which 93.7% were moderate and major (III–V) injuries. This is consistent with the 92.4% success rate in the systematic review conducted by Saqib *et al.*^10^

Current protocols advise close observation of patients undergoing NOM for liver GSW in the intensive care unit, consisting of continuous monitoring of vital signs, serial abdominal physical examination and serial laboratory measurements.^13,14,19–22^ All our patients in the NOM group were admitted to an intensive care unit or remained in the emergency room with continuous monitoring and haemoglobin control in the first 24 hours following admission.

The Pan-American Society of Trauma Consensus^23^ of 2019 states that patients with suspected penetrating liver injury with stable vital signs and absence of peritonitis should undergo CT scan to confirm peritoneal violation, determine the missile trajectory, classify the liver injury according to the AAST Injury Scoring Scale, identify potential associated intra-abdominal injuries, and detect active bleeding, false aneurysm or arteriovenous fistulas that could benefit from angioembolisation.^1,3,6,9,13–16^ CT scan with intravenous contrast is reported to have a 90.5% sensitivity and 96% specificity for the identification of GSW requiring surgical exploration.^1,24^ The presence of pneumo- or retropneumoperitoneum, free intraperitoneal fluid in the absence of solid organ injury, missile tract adjacent to hollow viscus with surrounding haematoma and high-energy GSW such as military and hunting weaponry are absolute contraindications for NOM.^1^ However, the need of mandatory imaging is not yet a consensus, and several authors advocate the decision to proceed with NOM should rely primarily on serial clinical examination.^1,3^

Previous authors^3,13^ suggest associated brain or spinal cord injuries are potential contraindications to NOM of GSW to the abdomen as regular physical examination may be compromised. In our study, in addition to the careful selection of patients based on specific criteria, we concluded the attending surgeon’s experience was also a determining factor for indicating NOM.

Most NOM failures (80.5%) occur in the first hours after hospital admission, leading to immediate laparotomy.^13,16,25,26^ We recorded two failures of NOM which prompted surgical exploration due to clinical deterioration during serial evaluation.

Complications of NOM of GSW to the right thoracoabdominal region consist mainly of missed injury to hollow viscus and associated injuries to the diaphragm and pleura.^5,14^ Al Rawahi *et al*^5^ reported the incidence of pulmonary complications is nearly 16%, while biliary complications can occur in up to 6.3% of cases. In our study, non-liver-related complications were prevalent in both groups, though no statistically significant difference was observed. Empyema was treated by thoracotomy in five NOM patients (16%), whereas no patient in the OM group required pulmonary decortication. This is likely due to the opportunity of clot evacuation through a diaphragm laceration during surgical exploration.

Liver abscesses are an infrequent complication, with an incidence of 1.2 to 3% and a mortality rate of up to 22%, as shown in the literature review by Dandin *et al.*^27^ These events are better characterised by contrast-enhanced CT scan and the treatment includes antibiotics, percutaneous drainage, laparoscopic drainage or open surgery.^13,16,27^ Two of our patients in the NOM group developed liver abscesses, which resolved after antibiotics or minimally invasive interventions. In the OM group, one patient required a second laparotomy to resolve the infection. By all means, the incidence of complications in patients undergoing NOM is reported to be similar or even lower than mandatory surgical exploration, given the 22–41% morbidity related to laparotomy.^5^

In the systematic review conducted by Al Rawahi *et al*,^5^ the LOS among previous studies of NOM of GSW to the abdomen averaged 2.3 days, extending to 6 days for those who developed extra-abdominal-related complication. In our series, LOS was similar between groups, with a median of seven days, which was likely owing to the associated pleuropulmonary injuries and their complications, precisely as Starling *et al*^20^ observed.

In conclusion, there is acceptable evidence that patients sustaining GSW to the liver who are evaluable upon abdominal examination, haemodynamically stable and without diffuse abdominal pain are candidates for NOM. Proper indications should be guided by clinical serial examination and CT scan findings. This study has limitations that deserve mention. Being single-centred, registry-based and non-randomised precludes to fully controlling for confounding. The limited sample size also has limited statistical power for some comparisons, and the assessment of long-term complications was limited by the short follow-up period. On the other hand, the cohort design was valuable for assessing the benefits of the surgical and non-surgical management of liver GSW, considering the daily routine of a trauma referral centre. The assessment of prognosis in patients submitted to both management strategies and its clinical outcomes may be considered a major strength of our study, thus contributing to the low but growing acceptance of selective NOM of GSW to the abdomen.

## Conclusions

Considering that in-hospital death, complications and LOS were similar between groups, this study suggests that NOM of GSW to the liver in selected patients is safe and effective, even for higher-grade injuries. This approach must be proposed within specific criteria, such as haemodynamic stability, abdominal CT scan and serial physical examination.

## Declarations

### Data availability statement

The datasets generated and analysed during this study are available from the corresponding author upon reasonable request.

### Funding statement

No funding to declare.

### Conflict of interest disclosure

All authors have no conflicts of interest to disclose.
